# Case report of snaring-assisted TAVR under cerebral embolic protection: the “Chaperone” with “Top Hat” technique

**DOI:** 10.3389/fcvm.2023.1213817

**Published:** 2023-10-20

**Authors:** Massimo Medda, Francesco Casilli, Marta Bande, Maurizio Tespili, Francesco Donatelli

**Affiliations:** ^1^ Clinical and Interventional Cardiology Unit, Cardio-Thoracic Center, IRCCS Ospedale Galeazzi-Sant'Ambrogio, Gruppo San Donato, Milan, Italy; ^2^ Clinical and Interventional Cardiology Unit, Cardio-Thoracic Center, Istituto Clinico Sant'Ambrogio, Gruppo San Donato, Milan, Italy; ^3^ Department of Cardiothoracic Center, IRCCS Ospedale Galeazzi-Sant'Ambrogio, University of Milan, Milan, Italy

**Keywords:** aortic valve stenosis, snare catheter, chaperone technique, transcatheter aortic valve replacement (TAVR), cerebral protection devices

## Abstract

Symptomatic severe aortic stenosis (AS) in patients with intermediate-to-high surgical risk is currently being treated with transcatheter aortic valve replacement (TAVR). We present a case of a TAVR in a severe calcific AS with porcelain aorta and ‘gothic’ aortic arch. Pre-operative thoraco-abdominal computed tomography angiography showed also severe calcification at the sinotubular junction with protruding huge calcified nodules extending in ascending aorta and multiple calcific stenosis of both iliac-femoral vessels, severely tortuous. The choice of the interventional access was not easy and the high risk of an acute intra-procedural brain event guided the procedural planning. To our knowledge, this is the first case of TAVR with complete cerebral protection with Triguard system device and ‘snaring-assisted’ valve advancement.

A 84-year-old woman was admitted for effort dyspnea (NYHA class II-III) in severe aortic stenosis (peak/mean gradient 107/61 mm Hg, aortic valve area 0.5 cm^2^) with high surgical risk (STS PROM 8%). Pre-operative computed tomography angiography showed the aortic valve and the sinotubular junction severely calcified, multiple protruding calcific nodules of ascending aorta, “gothic” aortic arch (AA) and multiple calcific stenosis of both iliac-femoral vessels, severely tortuous (Figures 1, 3–4, Supplementary Figure S1). We planned a transfemoral implantation of a 29 mm self-expandable Evolut R valve (Medtronic, Minneapolis, Minnesota, USA) under complete cerebral protection (CP) with TriGuard™ system and delivery advancement “snaring-assisted” (Figures 2, Supplementary Figures S3–S5) (1, 2). The placement of a CP system has been planned because the patient had a history of previous stroke, due to the presence of severe calcifications of the ascending aorta, of the sino-tubular junction and of the aortic valve (in addition of suspected calcific bridge between the left coronary cusp and the right coronary cusp) (Figures 3, 4). Based on the valve anatomy, the presence of very elliptical aortic annulus and LVOT and the huge calcifications of the sinotubular junction, we considered that the implantation of a self expanding valve was the most correct choice. The snaring procedure of self-expanding THV has been planned to anticipate traumatic contact with the aortic wall calcifications and to easier navigate with THV in the “gotic” and calcific AA (Figures 1, 3, Supplementary Figures S3–S5). The procedure required the management of 5 vascular accesses: 1 radial access + 2 femoral accesses + 2 ancillary femoral accesses (Figure 1, panels B-C). In order to guide the advancement of the transcatheter heart valve (THV) through the AA we preventively inserted a 20-mm AndraSnare catheter (Andramed, Reutlingen, Germany) from the contra-lateral femoral artery.

**Figure 1:**
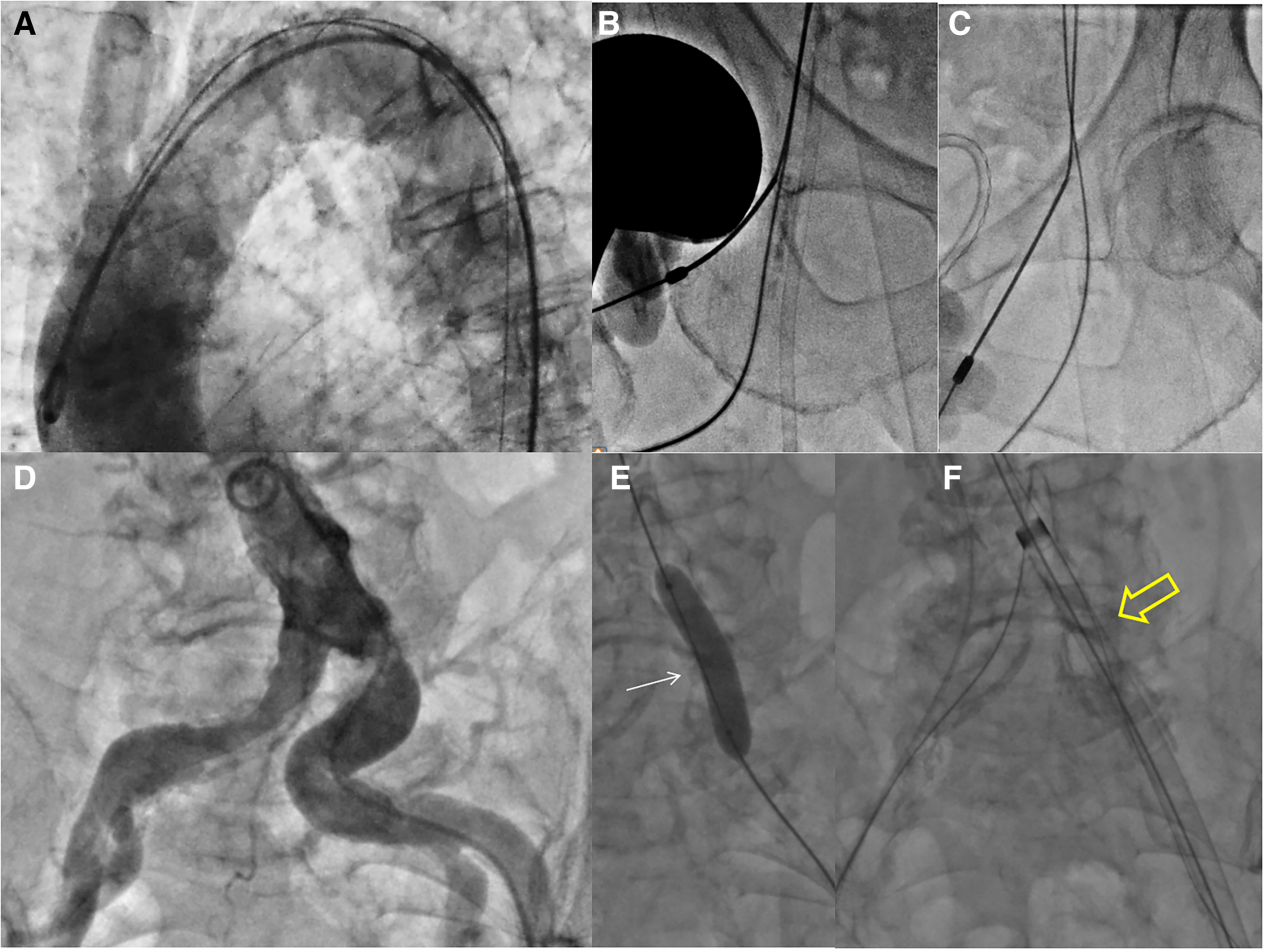
(**A**) Basal aortography showed the diffuse aortic wall calcifications and very angulated aortic arch. (**B**–**C**) Double bilateral femoral punctures. (**B**) common and superficial right femoral sheaths, respectively for the cerebral embolic device (8F) and 20 mm snare (5F). (**C**) common and superficial left femoral sheaths, respectively for interventional access and ipsilateral femoro-femoral “wire protection” (5F). (**D**) Aortography showed multiple calcific stenosis of both iliac-femoral arteries. (**E**–**F**) The 18F Cook Introducer (white arrowhead) was advanced via a brachiofemoral “through-and-through wire technique” after dilatation of ostial left common iliac artery stenosis with a semi-compliant 8 mm balloon (white arrow).

**Figure 2:**
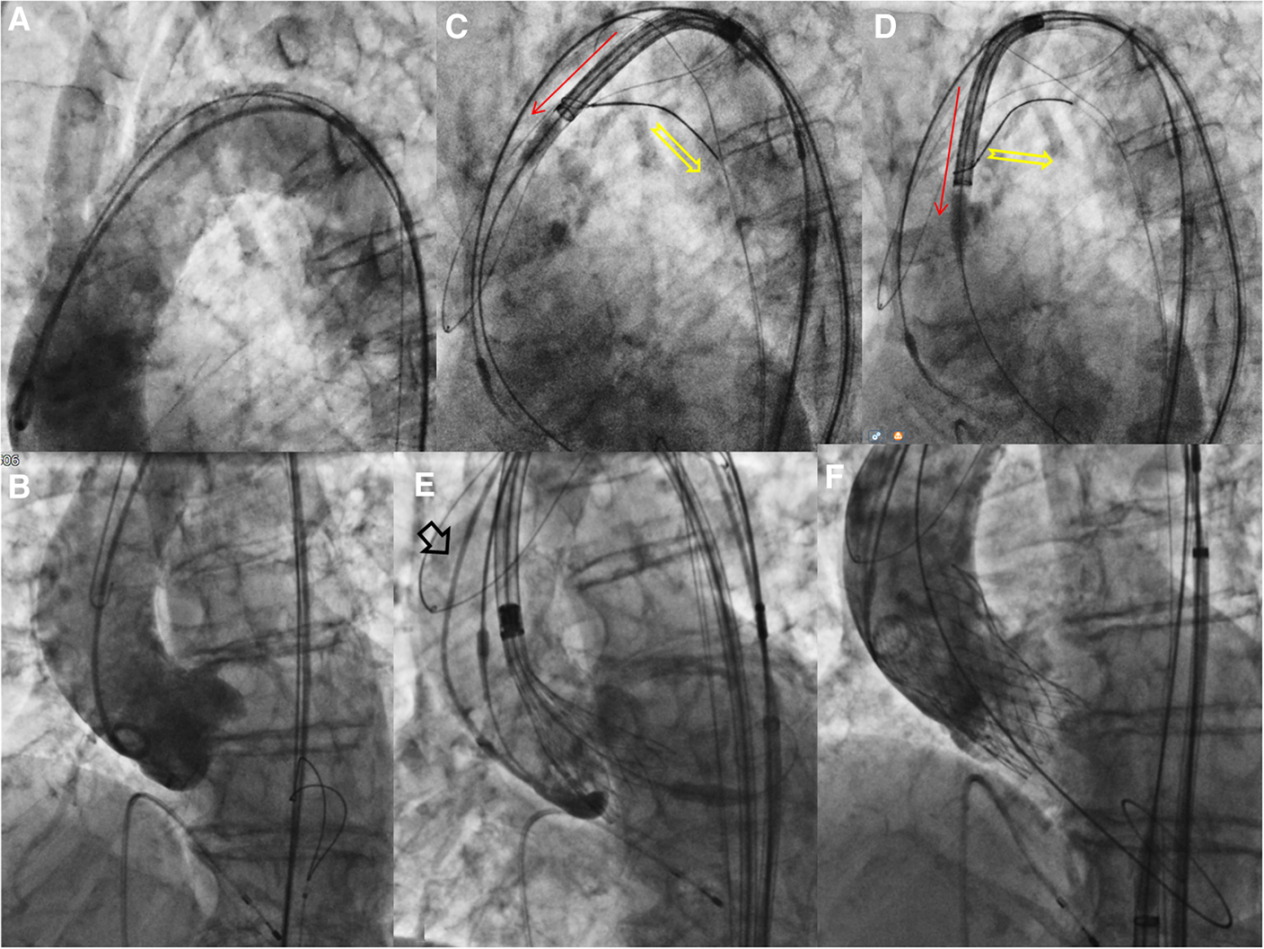
Procedural steps -2. (**A**) Aortography showed calcific very angulated aortic arch. (**B**) Aortography in the “virtual basal plane” view with pre-mounted snare. (**C,D**) The snared valve is pulled (yellow arrow), detached from the aortic wall under complete cerebral protection and **(E)** advanced through ascending aorta. (**F**) The final angiography documented a good result without significant paravalvular leak.

**Figure 3:**
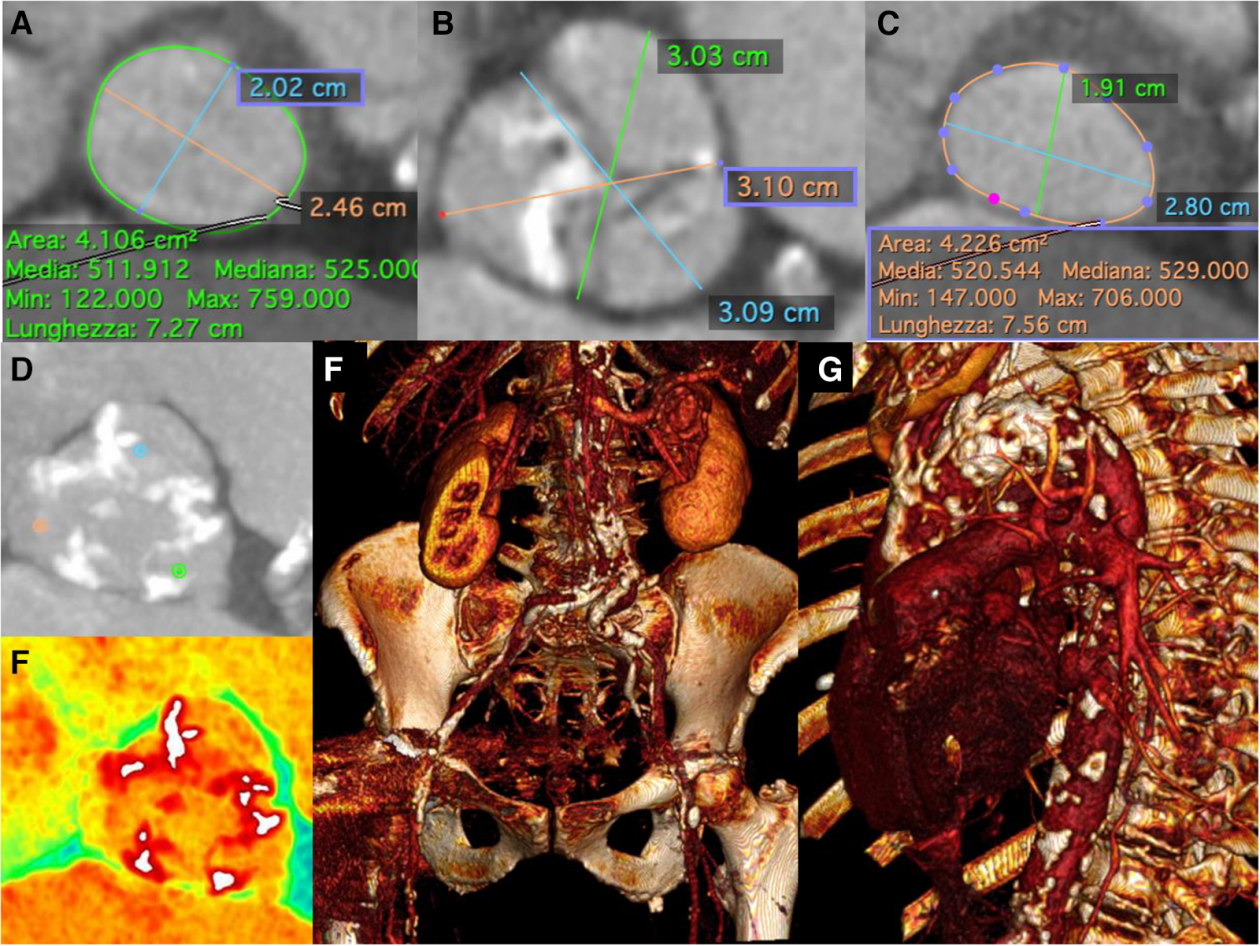
Pre-Procedural multislice computed tomographic evaluation. Pre-operative thoraco-abdominal computed tomography angiography showed (**A**) an aortic annulus perimeter of 72.7 mm (area of 410,6 mm^2^), (**B–D,E**) an aortic valve severely calcified, and (**C**) a left ventricular outflow tract (LVOT) perimeter of 75.6 mm with elliptical shape (area of 422,6 mm^2^), (**F**) “gothic” and calcific aortic arch and (**G**) multiple calcific stenosis of both iliac-femoral vessels, severely tortuous.

**Figure 4:**
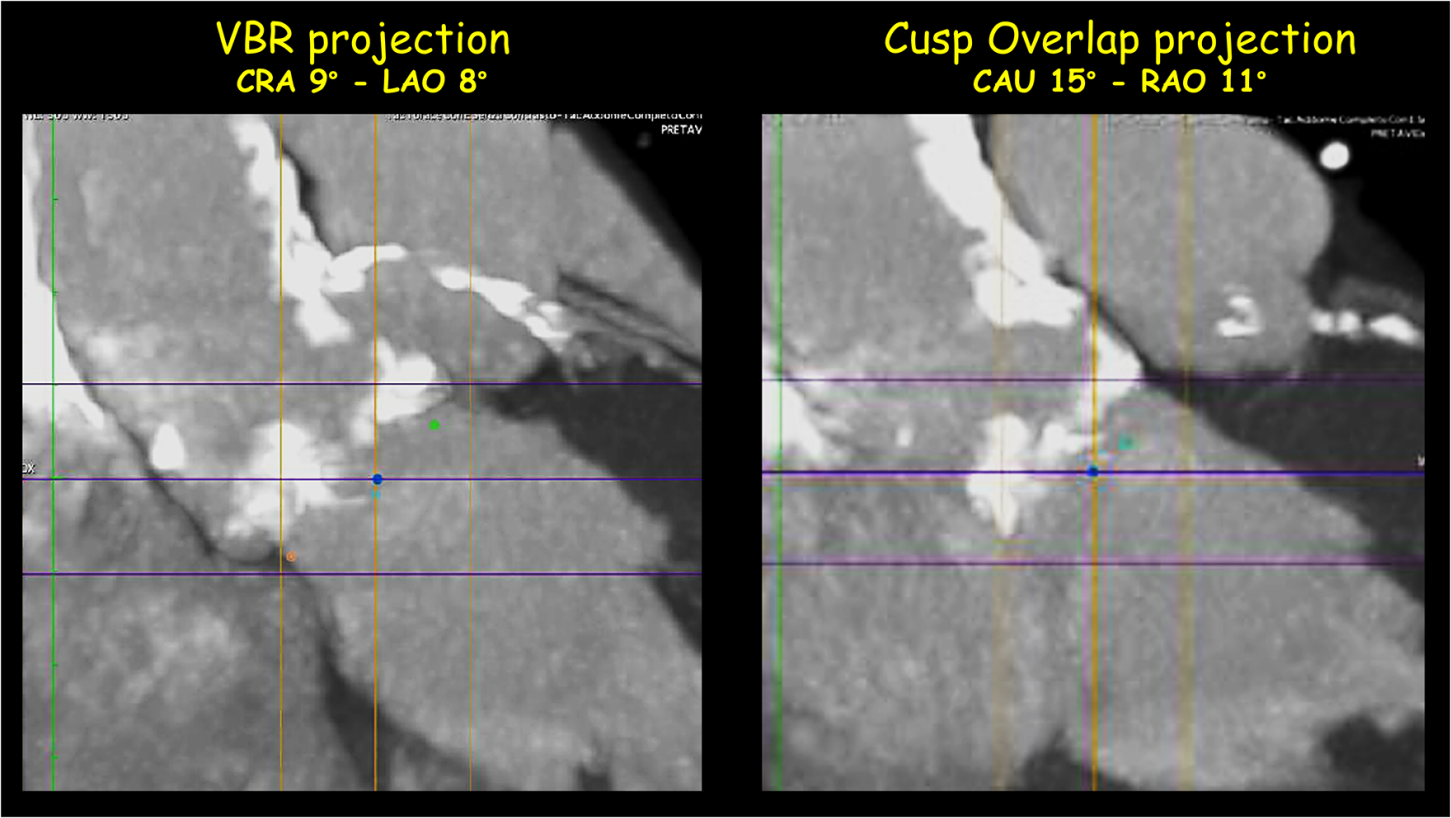
Pre-Procedural multislice computed tomographic evaluation: aortic calcifications. MIP-reconstruction of the thoraco-abdominal CT angiography showed the massive calcifications of the ascending aorta and the sino-tubular junction.

The THV was advanced on the snared guidewire, and the tip of the THV was ensnared and then pulled in order to be detached from the calcified wall of the ascending aorta, allowing the valve to be steered (“Chaperoned”) through them moreover without any interference with the CP system (“Top Hat”) (Figure 2) (Supplementary Movies S1–S4). There were no complications related to the snaring of THV, conversely the snaring maneuver has facilitated the “atraumatic” transit of the THV into the aortic arch. At the macroscopic analysis of cerebral protection device (Triguard) there were also present small debris of calcium (Supplementary Figure S2). Finally no complications related to the vascular accesses occurred. On the basis of our experience the risk-benefit balance of implementing cerebral protection with device (and specifically with Triguard) needs to be deeply evaluated because it requires a great care: (a) to avoid uncontrolled movement of the device once opened, (b) to avoid aggressive contacts with the aortic wall during complex maneuvers (c) the CP system requires a dedicated vascular femoral access (8F). To our knowledge, this is the first case of snaring-assisted TAVR under complete cerebral embolic protection with TriGuard™ system.

## Data Availability

The raw data supporting the conclusions of this article will be made available by the authors, without undue reservation.

## Supplementary material

Supplementary Figure S1

Pre-Procedural Multislice Computed Tomographic Evaluation: iliac-femoral arteries. Pre-operative thoraco-abdominal computed tomography angiography showed multiple calcific stenosis of both iliac-femoral vessels, severely tortuous

Supplementary Figure S2

Calcium debris captured by CP System. The figure highlights the calcium debris adhering to the Triguard system once the procedure has been completed and the protection system has been closed and removed from the patient.

Supplementary Figure S3

Procedural planning -1. Schematic representation of the snared valve detached from the aortic wall under complete cerebral protection and advanced through ascending aorta.

Supplementary Figure S4

Procedural planning—2. Schematic representation of the “snare-assisted” valve advancement through ascending aorta and “gothic” aortic arch.

Supplementary Figure S5

Procedural planning—3. Schematic representation of the deployment and opening of the THV under the cerebral protection (“Top Hat”) after snare releasing.

Supplementary Movie S1

Under fluoroscopic guidance, the TriGuard™ system is positioned in the ascending aorta and aortic arch to cover the ostia of the innominate, the left common carotid and the subclavian arteries.

Supplementary Movie S2

The snared valve was advanced into the aortic arch and meantime pulled, thus detaching it from the aortic wall (and from the cerebral protection system) and obtaining a deflection of approximately 180 degrees of the delivery system.

Supplementary Movie S3

Aortography of deployment in “coplanar view” documenting a correct valve position.

Supplementary Movie S4

Final aortography showed the correct implantation of an 29 mm Evolut R valve without significant perivalvular leak.
