# Supplementary figures and images for: Case report of snaring-assisted TAVR under cerebral embolic protection: the “Chaperone” with “Top Hat” technique
Source: Front Cardiovasc Med. 2023 Oct 20;10:1213817. doi: 10.3389/fcvm.2023.1213817 (PMC10623418; doi:10.3389/fcvm.2023.1213817)

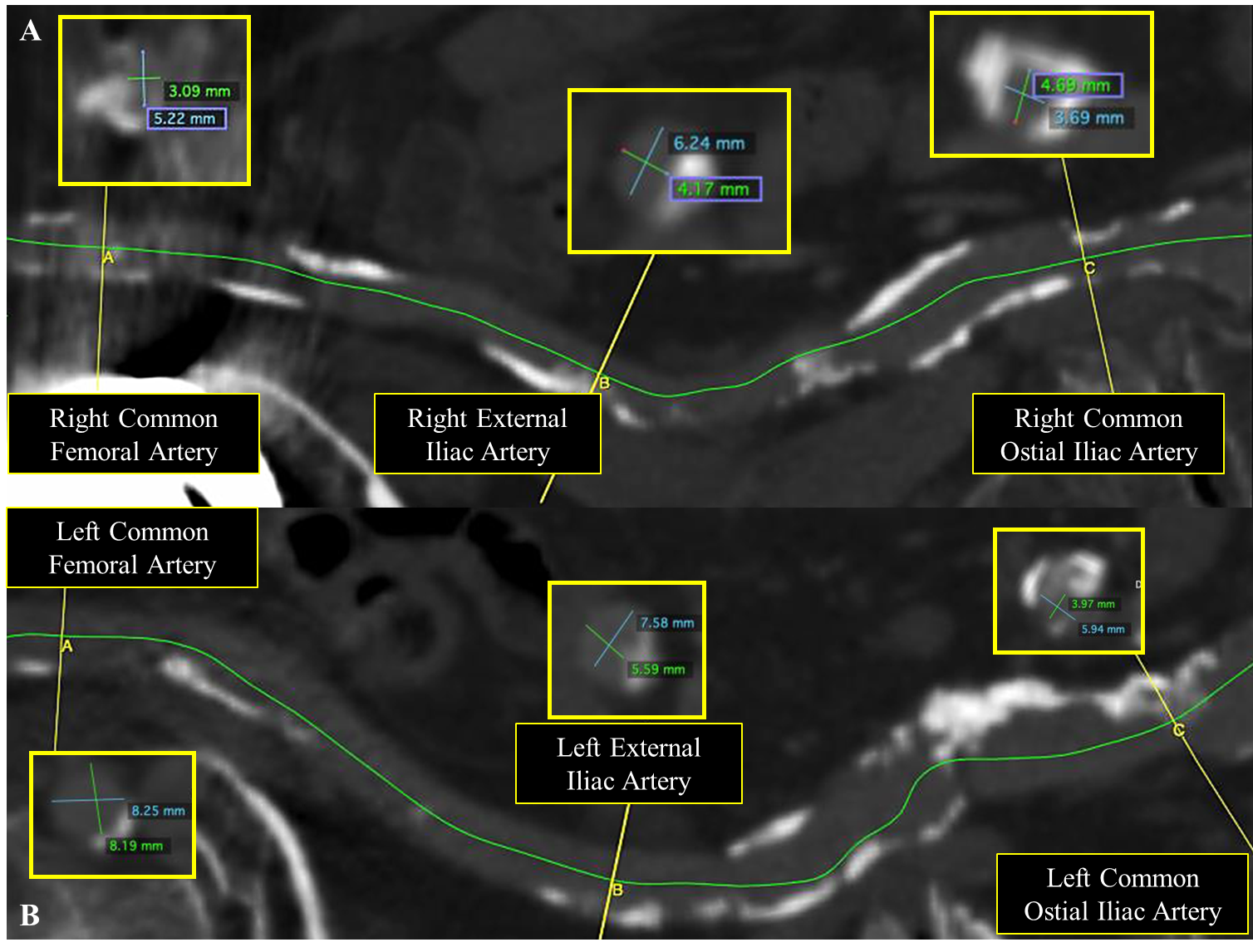

Supplement: Supplementary file 1 [file Image1.tif]

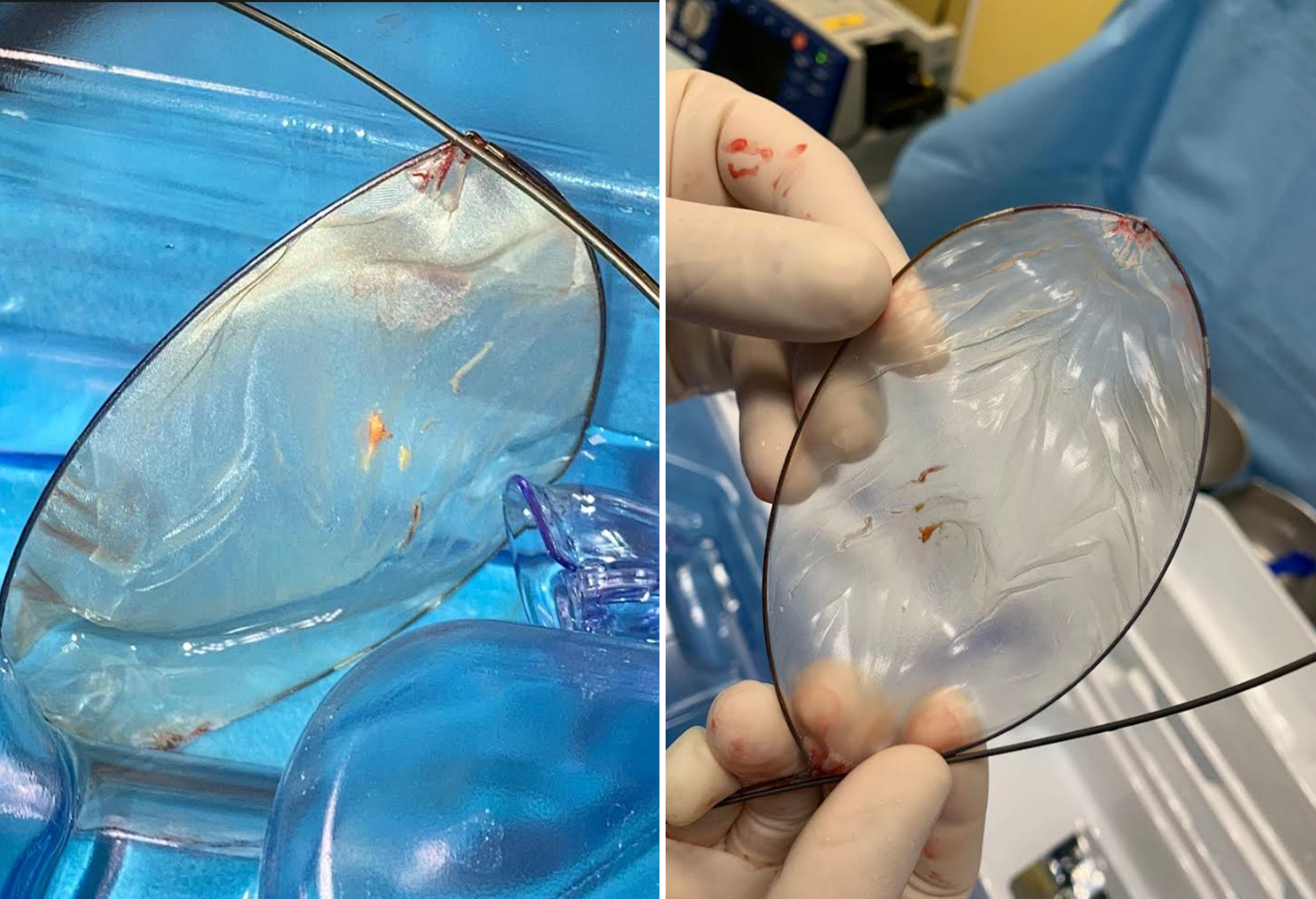

Supplement: Supplementary file 2 [file Image2.tif]

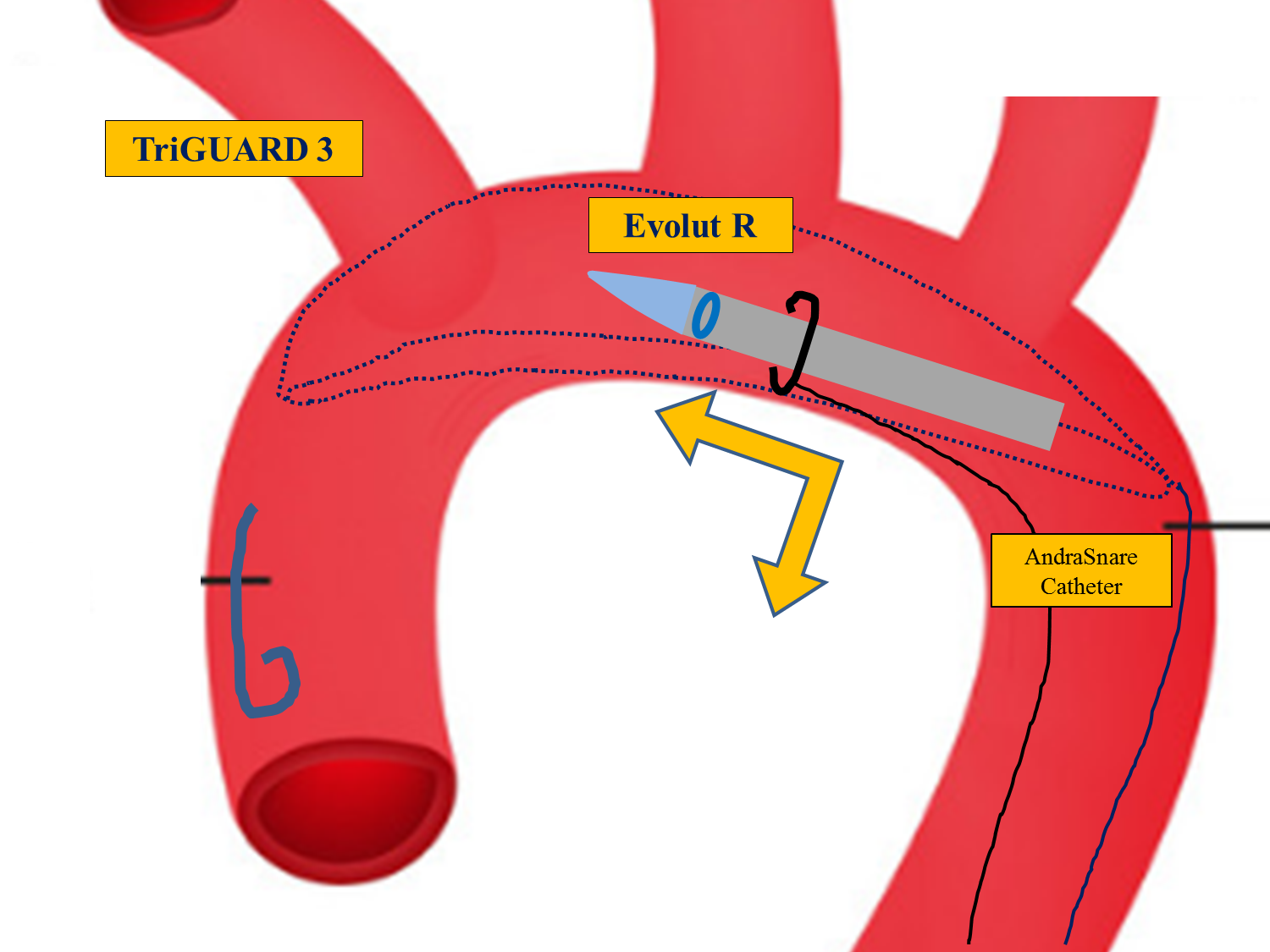

Supplement: Supplementary file 3 [file Image3.tif]

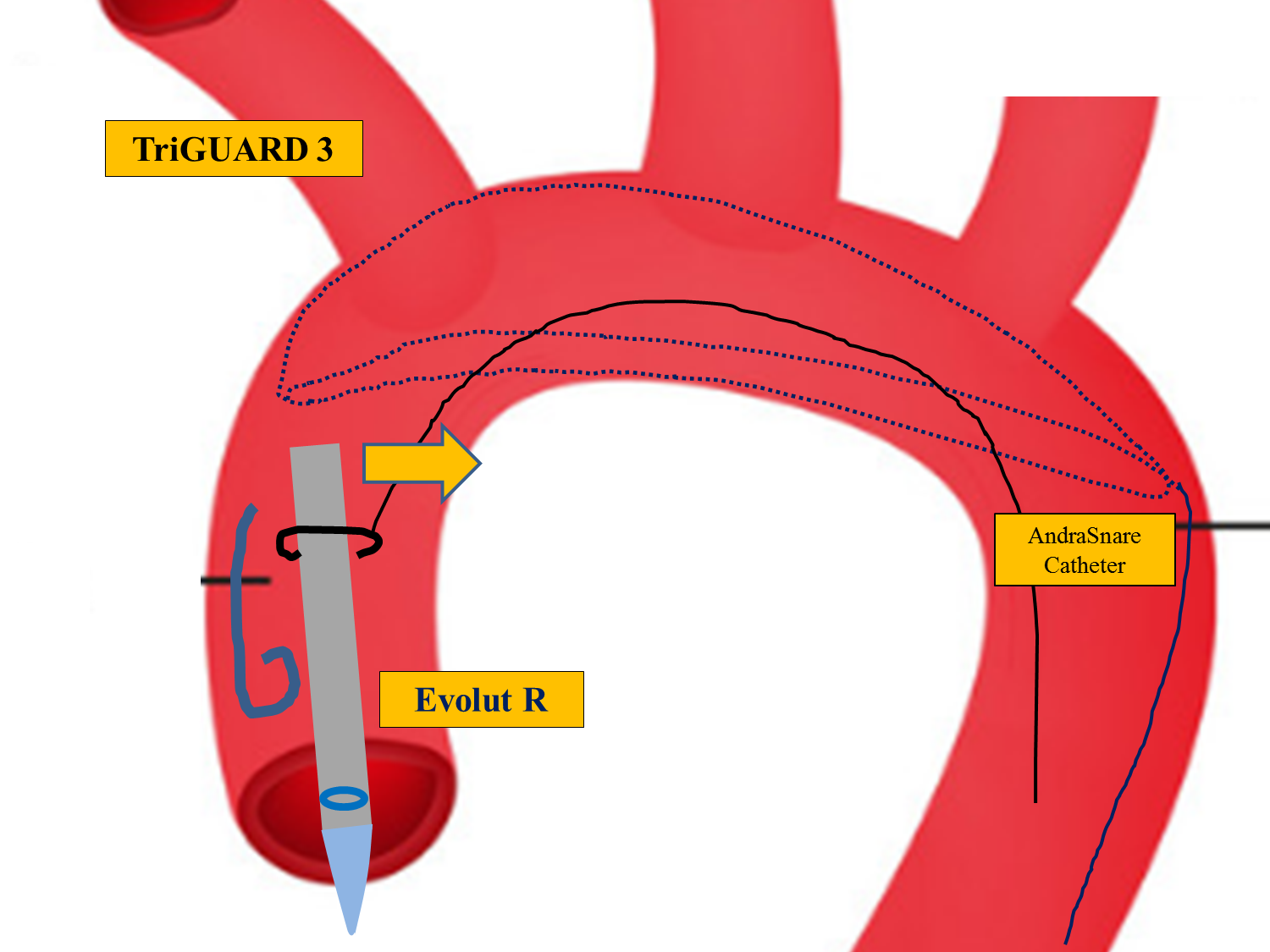

Supplement: Supplementary file 4 [file Image4.tif]

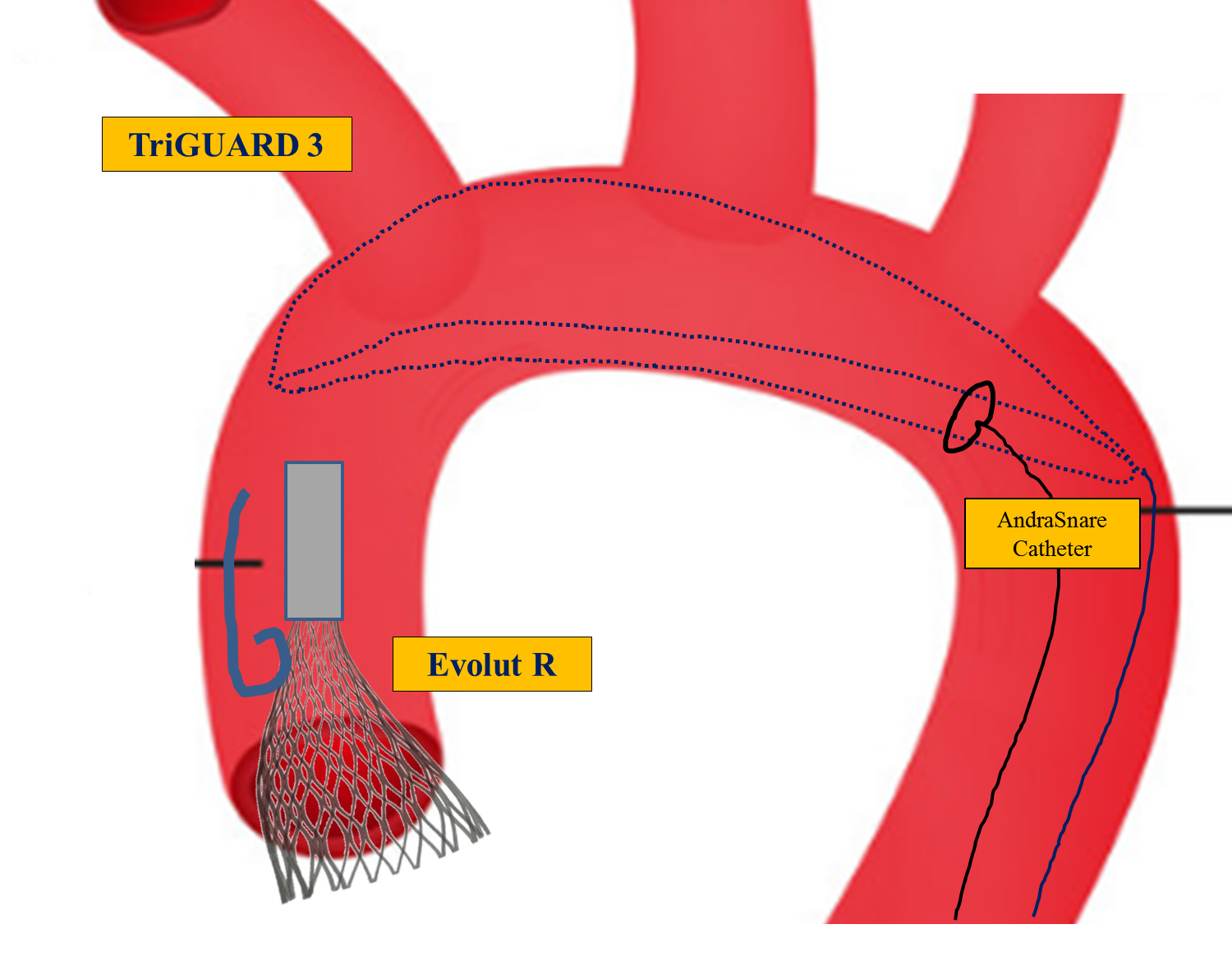

Supplement: Supplementary file 5 [file Image5.tif]
